# Quality Characteristics of Raspberry By-Products for Sustainable Production

**DOI:** 10.3390/foods13101436

**Published:** 2024-05-07

**Authors:** Audrone Ispiryan, Ingrida Kraujutiene, Jonas Viskelis

**Affiliations:** 1 Department of Food and Agrotechnology, Kauno Kolegija Higher Education Institution, Pramones Pr. 20, LT-50468 Kaunas, Lithuania; ingrida.kraujutiene@go.kauko.lt; 2 Institute of Horticulture, Lithuanian Research Centre for Agriculture and Forestry, Kauno Str. 30, LT-54333 Babtai, Lithuania; jonas.viskelis@lammc.lt

**Keywords:** raspberry seeds, sustainable products, nutrient content, micro- and macro-components, by-products, food waste valorization, functional food

## Abstract

Raspberry seeds are a by-product of berries, both from their primary processing, such as in juice production, and secondary processing, such as in oil extraction. These seeds contain plenty of valuable components such as crude fiber, proteins, fats, and vitamins. Quality characterization is the initial step toward using these seeds as a sustainable and functional food. The aim of studying raspberry seeds’ quality profile, both before oil extraction and after different processing methods (supercritical CO_2_, subcritical CO_2_, cold pressing, and hexane solvent), is to point out the benefits of this by-product and to raise consumer awareness about their health and well-being benefits. This study provides evidence that raspberry seeds have good physical parameters for use in other products as a functional food enrichment ingredient, such as in baked goods, offering considerable health benefits due to their high nutrient content. The weights, peroxide values, moisture content, nutritional energy values, and colors were determined before oil extraction to give initial seed values. The nutrient content and amounts of macroelements, P, K, Ca, and Mg, as well as microelements, B, Zn, Cu, Fe, and Mn, were determined in the tested variety ‘Polka’, both before and after oil extractions and using different methods. The raspberry seeds’ moisture was 9.2%, their peroxide content was 5.64 mEq/kg, their nutritional value was 475.25 Kcal., and their total weight was 2.17 mg (1000 units). The seeds contain 7.4% protein, 22.1% crude fiber, 11.0% crude fat and oil, and 2.8% sugar. We determined how different oil extraction methods influence the nutrient, micro-, and macro-component values. We concluded that the seeds contained the highest manganese (45.3 mg/kg), iron (29.2 mg/kg), and zinc (17.4 mg/kg) contents and the lowest content of copper (5.1 mg/kg). This research shows that raspberry seeds represent a potential natural food ingredient, and after oil extraction with subcritical or supercritical CO_2_ or cold pressing, they can be used as a sustainable and functional food.

## 1. Introduction

Raspberries (*Rubus idaeus* L.) constitute a rich source of bioactive compounds [1,2,3]. They contain diverse nutritious components that are important for leading a healthy lifestyle [4]. The production of value-added products from raspberry waste streams may also offer significant well-being benefits. In the production of berry juices, the liquid is separated from the waste containing the seeds. A value-added product, seed oil, can be obtained, leaving seed flour. Both product streams are gaining more and more attention, constituting a small but growing segment of the market [5]. The contemporary challenges faced by industries, scientists, and humanity as a whole are reducing food waste, optimizing production processes (minimizing sidestream production), and valorizing residues to obtain valuable and sustainable products. It is important to ensure that all by-products constitute a resource/raw material that can be further used [6].

Such seeds contain plenty of valuable components, such as fibers [7,8], proteins [9,10], and micro- and macro-components [11,12,13]. These researchers suggest that raspberry seeds may contain approximately 25–30% fiber by weight. This fiber content contributes to digestive health, regulates bowel movements, and may help lower cholesterol levels. The protein content of raspberry seeds averages around 22–25% of their dry weight and is rich in essential amino acids, which are the building blocks of proteins necessary for various physiological functions in the body.

Paying attention to new information about the various sustainability challenges that arise is paramount to adding value to fruit and berry waste in order to extract health-promoting food. However, researchers have noticed that seed valorization is at an early stage of development and that essential elements are yet to be clarified in order to assess their viability [14]. Nevertheless, such valorization would constitute a step in the right direction for the sustainable intensification and diversification of the global food base [15].

Raspberry seeds (RSs) represent a potential source of natural food ingredients. For example, epidemiological studies have linked the consumption of these seeds to improvements in the symptoms of a number of diseases, such as atherosclerosis, diverticulosis, colon cancer, and more. In recent decades, scientific research into reducing the incidence of these diseases has intensified; food manufacturers are increasingly using waste from primary or secondary food production processes, looking for opportunities to enrich food with plant fibers, to improve processes, their safety, and efficiency, and to make the product more appealing to the end consumer.

Currently, this source is not thoroughly exploited, although there are significant opportunities for the food industry in this area. The exploitation of raspberry seeds during fruit processing, both as a source of functional compounds and for application in food is a promising field which requires additional research [16]. It should be noted that the quality characteristics of raspberry seeds before oil pressing or after different pressing methods have not been reported. Sigh at al. recently reported that knowing the impacts of food processing on nutritional contents allows people to make more educated dietary choices [17].

Moreover, in recent times, technologies have improved significantly, and oil producers use a myriad of techniques that have various effects on raw materials during production: adjusting temperature, pressure, ultrasound, etc. In this way, they can optimize the sustainability of raspberry processing, which can, in turn, improve raspberry seed yield, as well as their nutritional and functional properties. The use of such products can contribute significantly to improving people’s health. Processing technologies and parameters can significantly also impact the quality characteristics of berry seeds. It can break down vitamins and lead to the degradation of oil in the seeds. It might affect the flavor, aroma, and nutritional value. Lower or controlled temperatures can help to maintain their nutritional quality. The intended use of the seeds, and the desired to create product, must indicate how to optimize proper and control processing conditions. It even shows which processing technology must be chosen to preserve the required nutritional value, texture, color, and overall quality of berry seeds.

The aim of this research was to collect data on the quality characterization properties of raspberry seeds, both before oil extraction and after different processing methods, and to point out the benefits of this by-product from sustainable processing. Ultimately, we wish to raise consumer awareness about the health benefits of raspberry seeds.

## 2. Materials and Methods

### 2.1. Plant Material

**Figure 1:**
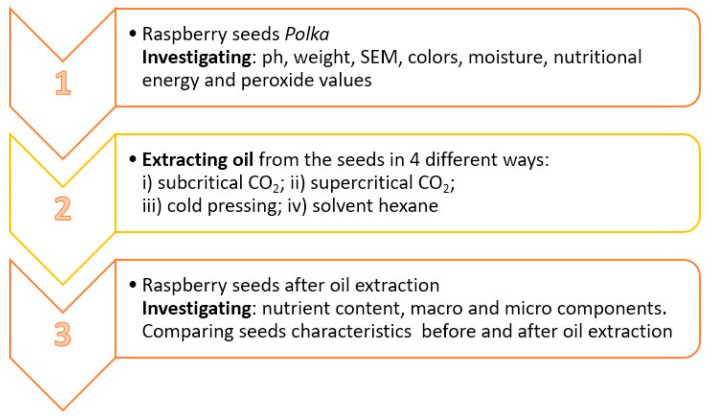
Methodological overview.

An overview of the methodology employed in the study is presented in Figure 1. 

### 2.2. Plant Material and Samples Preparation

The raspberry seed variety ‘*Polka*’ was obtained after industrial juice production. The seeds were air-dried at room temperature (+22 °C) with constant stirring to reduce the moisture to 9.2%. The seeds were ground. Five replicates of 100 seeds were counted and weighed. In the second stage, we evaluated the seeds both before and after oil extraction via 4 different methods: extraction with subcritical CO_2_, extraction with supercritical CO_2_, solvent (hexane) extraction, and cold pressing. 

Cold pressing was carried out with the cold pressing Machine PR-H100/1 (1Head) (Oil press GmbH & Co. KG, Reut, Germany) at a speed of 10 Hz (8 RPM), capacity of 2.38 kg/h, and yield of oil of 0.3 kg/h.

A Soxhlet apparatus is used for solvent extraction; seeds were extracted with n-hexane. The extraction took about 48 h. After extraction, the solvents were removed in a vacuum rotary evaporator at a temperature of 40 °C to a constant mass. 

Extraction with subcritical and supercritical carbon dioxide: Dried raspberry seeds are ground with an ultracentrifugal mill ZM200 (Retsch, Haan, Germany) using a 0.2 mm sieve. The oil was extracted with subcritical CO_2_ at a pressure of 5 MPa and a temperature of 10 °C for 16 h with a subcritical extractor Eco Extractum, Lithuania. The supercritical extraction experiments were carried out using supercritical fluid extractor SFT-150 (Supercritical Fluid Technologies, Newark, DE, USA). Each extraction was performed using 500 g of ground dried raspberry seed sample. Each sample was loaded into 150 mL thick-walled stainless cylindrical extractor vessel with 5-micron frits. The temperature (60 °C) of the extraction vessel was controlled by a surrounding heating jacket. The volume of CO_2_ consumed was measured by a gas flow meter Gallus 2000 (Schlumberger Industries, Guebwiller, France) and expressed in standard liters per minute (SL/min). Flow rates were 1.4 SL/min. The process consisted of static (120 min) and dynamic (300 min) extraction steps. The static extraction time was included in the total extraction time of 650 min.

### 2.3. Determination of Weight

The thousand-seed weight determined by the traditional methodology described in the Rules for Seed Testing and International Rules for Seed Testing [18] followed three steps: (1) manual count of eight replications of 100 seeds of the pure seed portion; (2) weighing of samples on a scale (0.001 g); and (3) calculation of thousand-seed weight. The TSW was calculated in a data spreadsheet (Microsoft Excel 365) by filling cells in a pre-established table, which was obtained by estimation of the weight in grams of one thousand seeds.

### 2.4. Determination of Moisture

Seed moisture was determined by weighing a sample before and after drying at a constant temperature for a set period of time (Shimadzu Moisture Analyser MOC63u, Shimadzu corporation, Tokyo, Japan). Raspberry seeds of the tested variety ‘*Polka*’ were examined using images obtained from Scanning Electron Microscopy (SEM) FEI Quanta 200 FEG (FEI Company, Hillsboro, OR, USA). Images were taken at a scale bar of 1.00 mm and 500 μm and were analyzed in low-vacuum mode operating at 3.0 kV using an LDF detector.

### 2.5. Determination of Peroxide Value

The peroxide value was evaluated using the classical AOCS method (AOCS Official Method Cd 8b-90). The acetic acid–isooctane method was used to determine the PV (AOCS 1996a). This method determines all substances in terms of milli equivalents of oxygen per 1000 g of sample; this is achieved through the oxidization of potassium iodide under the test conditions. These substances are generally assumed to be peroxides or other similar products of fat oxidation. 

### 2.6. Color Measurement

The color coordinates of the raspberry seed samples in the uniform contrast color space CIE L*a*b* were measured using a MiniScan XE Plus spectrophotometer (Hunter Associates Laboratory, Inc., Reston, VA, USA). The parameters evaluated during reflected-color measurements were L*, a*, and b* (brightness and red and yellow coordinates according to the CIE L*a*b* scale, respectively), and color saturation (the chroma value) was calculated (*C* = (a*2 + b*2)1/2). The values L*, a*, b*, and C* were measured in NBS units. The NBS unit is a unit of the U.S. National Bureau of Standards and meets one color resolution threshold, i.e., the smallest difference in a color that can be captured by a trained human eye. Prior to each series of measurements, the spectrophotometer was calibrated with a light trap and a white standard with the following color coordinates in the XYZ color space: X = 81.3, Y = 86.2, and Z = 92.7. The value of L* indicated the ratio of white to black, the value of a* indicated the ratio of red to green, and the value of b* indicated the ratio of yellow to blue. Five replications were taken for the analysis. The color coordinates were processed by the Universal Software V. 4-10.

### 2.7. Determination of the Amount of Macro- and Microelements

The microelements were determined based on the following regulations: moisture—directive 71/392/EEC; nitrogen and proteins—72/199/EEC; crude fiber content—directive 73/46/EEC; crude fat and oil content—directive 71/393/EEC; phosphorus (P)—directive 71/393/EEC, potassium (K) and calcium (Ca)—directive 71/250/EEC, magnesium (Mg)—directive 71/46/EEC, and boron (B), zinc (Zn), copper (Cu), iron (Fe), and manganese (Mn)—LST EN 15510:2017. Macronutrient contents were expressed in mg/ g 1, and micronutrients were expressed in g 1 dry weight.

### 2.8. Statistical Analysis

All the experiments were carried out in triplicate. MS Excel 365 (Baton Rouge, LA, USA) and IBM SPSS Statistics, Windows (Redmond, WA, USA) software packages were used for statistical analyses. One-way analysis of variance (ANOVA) along with the post hoc Tukey’s HSD test was employed for statistical analysis. Differences were considered to be significant at *p* < 0.05.

## 3. Results and Discussion

### 3.1. Raspberry Seeds Characteristics before Oil Extraction

The main quality characteristics such as the weight, peroxide value, moisture, nutritional energy value, and colors of the seeds before oil extraction are presented in Table 1. 

The data presented in Table 1 outline several key quality characteristics of raspberry seeds before oil extraction. Understanding each of these parameters is crucial for assessing the quality and potential uses of raspberry seeds in various applications. Raspberry seeds weigh 2.17 g/1000 seeds, which provides a baseline for the size and density of the seeds that is useful for processing and application considerations. The relatively light weight suggests that raspberry seeds are small, which might impact the efficiency of oil extraction and the texture in food products. The peroxide value is an indicator of the extent of lipid peroxidation or the rancidity of fats in the seeds. A value of 5.64 mEq/kg is relatively low, indicating that the seeds have a good level of freshness and minimal oxidative damage. This low peroxide value is beneficial for the shelf life and quality of the oil that can be extracted from these seeds, as higher values would suggest a higher degree of spoilage or degradation of the oil. The moisture content in seeds affects both their storage and processing characteristics. A moisture content of 9.2% indicates that the seeds are not overly dry or overly moist. This level is likely optimal for maintaining seed viability without encouraging microbial growth or spoilage. Proper moisture levels are essential for effective oil extraction and for ensuring the stability and longevity of the seeds during storage. The high caloric value of raspberry seeds (475.25 Kcal) reflects their rich nutrient composition, which is primarily from fats and proteins. This makes them an excellent energy-dense food ingredient that is suitable for nutritional supplements and health foods. The caloric content is particularly important for targeting dietary energy requirements in food product formulation where high-energy, nutrient-dense ingredients are valuable. 

Comparing with other studies, it is important to notice that we analyzed the weight of the *Polka* raspberry seeds. Moore et al. [19] have reported on the seed weights and sizes of economically important *Rubus* cultivars. The authors reported that seed and fruit weights are positively correlated. In their study, the seeds of seven different varieties weighed (1000 pieces) between 2.01 and 4.83 g. The average seed weight was 2.98 g. Hummer et al. [20] measured 43 *Rubus* seeds from different countries (Japan, JAV, Russia, etc.). Their weights ranged from 0.3 to 9.5 g, and the average seed weight was 1.72 g. 

The seed weights of European *Rubus* species ranged from 1.3 to 2.7 mg. Dimić et al.’s [21] weight test for 1000 raspberry (‘*Willamette*’ variety) seeds showed a significant difference in weight between 3.5 g (dry basis) for blackberry pomace and 1.5 g for raspberry pomace. However, we did not find weights for the Polka variety in the literature, so we can only compare our results with seeds of other varieties. Compared to the works above, our 1000 investigated seeds weighed 2.17 mg (mean of three replications), which is close to the highest value in Europe. In the future, it would be interesting to conduct research into how cultivation techniques simultaneously affect the seed and berry sizes.

Peroxide value is another important criterion in examining seed quality. The amount of peroxides shows the degree of fat oxidation. In general, this value should not exceed 10 mEq O_2_/kg in oil, but for some oils, it can be up to 20 mEq O_2_/kg. If these values are significantly exceeded, the oil becomes rancid. An example of this can be found in V. Van Hoed et al.’s [22] study, in which the authors found a peroxide value of 42.88 mEq O_2_/kg oil in raspberry seed oil. A scientific study by Raczyk et al. [23] showed the low quality of raspberry oils in terms of oxidative stability (high peroxide values). The peroxide value increased from 21.9 to 47.6 mEq O_2_/kg oil after 8 weeks of storage in a cool place. It would also be valuable to investigate the influence of the seed peroxide value on their preparation and technological process parameters, e.g., at what temperature the seeds are dried.

Compared to our study, these seeds had much lower values at only 5.64 mEq O_2_ /kg extract. This shows that fresh raspberry seed oil, when stored under the right conditions, should not have a bitter taste. As changes in the peroxide value during storage can be taken as one of the indicators of oxidative stability, it could be interesting to investigate how storage conditions and time influence seed and oil quality.

Moisture content is one of the most important factors that influences the quality and storage life of seeds. Seed moisture status is an important property that ultimately determines (i) whether they are metabolically active or quiescent; (ii) the relative vigor and viability loss rates; and (iii) whether they are alive or dead [24]. In our previous studies [25], raspberry seeds with 8.64% moisture content had a yield of 13.5% using solvent extraction, while Oomah et al. [26] reported that raspberry seeds with 13.6% moisture content had a yield of about 10.7%. In this study, the seeds had a moisture content of 9.2%. This shows that the seed is healthy and of a good quality. It is clear that the seed moisture content has an influence on the amount of oil extracted. Thus, scientific research to examine this influence would be relevant.

Affected by both the seed size and microstructure, seed hardness is also an important factor that influences milling energy and seed damage during storage. Hardness, defined as the force required to deform or crush the seed, only accurately describes the mechanical resistance of seeds that are the same size and shape [27]. Microstructural analysis provides detailed insights into the physical, biochemical, and nutritional characteristics of berry seeds, contributing to a comprehensive understanding of their quality and potential applications. Microstructural analysis contributes to the overall understanding of berry seed quality, providing the cellular structure as well as quality assessment, nutrient distribution, and quality control. By examining raspberry seed samples under a microscope, we identified that there are no impurities or contamination that may affect seed quality and safety. This allows ensuring the samples are of high quality.

**Figure 2:**
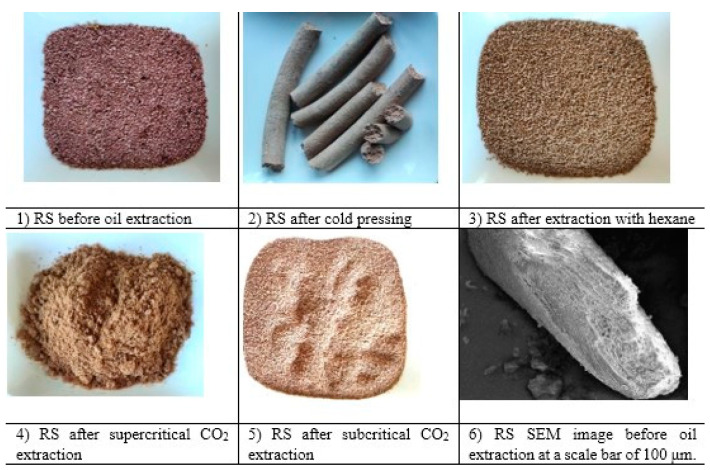
Raspberry seeds.

The microstructures of the raspberry seed were observed by taking SEM images; these can be seen in Figure 2. In the sample, the raspberry seeds had a quite regular, geometric appearance and a rough surface. The microscopic analysis results validate the significance of surface characteristics in determining the hardness and friction forces in plant material. This shows that making products from raspberry seeds can be difficult and that complex equipment is required.

The study results show that raspberry seeds before oil extraction are a viable source of nutritious oil and other by-products. The characteristics measured are indicative of seeds that can be stored, processed, and utilized effectively without immediate risk of spoilage or quality degradation. These baseline measurements provide crucial data for further research into improving raspberry seed processing techniques and developing new products that leverage their nutritional and functional properties.

### 3.2. Comparison of Raspberry Seed Characteristics before and after Oil Pressure

In the second stage, we analyzed raspberry seeds after oil extraction. Our research showed that different temperatures can affect the nutritional content of berry seeds by changing nutrient contents and especially the protein, crude fiber, fat and oil contents. This study revealed that the technical parameters of the processing processes have an unequal influence on the higher or lower amount of certain nutritional elements. A lower processing temperature does not necessarily lead to a better product quality. Raspberry seeds before oil extraction, after subcritical CO_2_ oil extraction, and after cold pressing are shown in Figure 2 below. We chose not to further analyze the seeds after hexane extraction because residues were found in them. Such a product would be neither sustainable or fit for human consumption. 

First of all, we paid attention to the colors, as these matter for the further production of food products. The most extensively researched and described of these is the use of seeds in confectionery and various baked goods. It is worth noting that although raspberry seeds are small, they should be ground to a flour for use in baked goods. Grinding the seeds before extracting the oil, using both supercritical and supercritical CO_2_ methods, can achieve higher yields, as we found in previous studies. Grinding the seeds before extracting the oil with hexane or using the cold method does not affect the yield, and the cold method even complicates the production process. After oil extraction via the CO_2_ subcritical method, the pulp of the seeds remains fluffy and can be used immediately in the production of baked goods. When the seeds are cold-pressed, they become compressed and very hard. In order to use this pomace in production, it is necessary to immediately crush it during pressing, without waiting for it to cool down, and then grind it. Raspberry seeds before and after oil extraction are shown in the Figure 2, and their colors are described in Table 2.

The data presented in Table 2 on the color characteristics of raspberry seeds before and after different oil extraction methods (CO_2_ subcritical, CO_2_ supercritical, and cold pressing) provide insightful information regarding how each extraction technique influences the color parameters of the seeds. The parameters measured, based on the CIELAB color space, include lightness (L*), red/green value (a*), yellow/blue value (b*), chroma (C*), and hue angle (*h*). The lightness (L*) value of the raspberry seeds before extraction is at 44.65. All extraction methods increase the lightness of the seeds, with cold pressing resulting in the most significant increase (55.66). This suggests that cold pressing might alter the seed’s surface texture or reflective properties more than CO_2_ extractions. Initially, seeds exhibit a moderate red/green value (12.65), but there is a noticeable decrease in the red/green value after all extraction methods, particularly with cold pressing (7.72). This reduction could indicate a loss or degradation of red pigments during extraction. Yellow/blue value of berry seeds before oil extraction stands at 13.26. Both CO_2_ extraction methods increase the yellow/blue value significantly, indicating a shift toward more yellowish hues post-extraction. Cold pressing results in a slightly higher increase (17.71) compared to the original, which could be due to different processing temperatures affecting pigment stability. Chroma starts at 18.35. All methods increase chroma, with the highest increase observed in seeds extracted using CO_2_ methods (around 22.29). This indicates a higher intensity of color post-extraction. The hue angle is initially at 46.23. There is a noticeable increase in the hue angle for all methods, most significantly in cold-pressed seeds (66.35). This change signifies a shift toward yellower tones. The variations in color parameters suggest that the method of extraction has a substantial impact on the color characteristics of raspberry seeds. The mechanical action in cold pressing might cause more disruption to seed pigments, altering lightness and hue more than the more controlled CO_2_ extractions. 

Changes in color characteristics can affect the aesthetic and sensory attributes of products made from these seeds. For example, lighter and more vividly colored seeds might be preferable for use in certain food products where visual appeal is important. The decrease in red/green values and increase in yellow/blue values might reflect biochemical changes in seed pigments during extraction, such as the breakdown of red anthocyanins and the relative stability or formation of yellow flavonoids. Understanding these color changes can help in selecting the appropriate extraction method based on the desired final product characteristics whether for aesthetic reasons in food products or for maximizing certain phytochemicals for health benefits. The results clearly show that different oil extraction methods can significantly influence the physical appearance of raspberry seeds, which could have practical implications for their use in the food and nutraceutical industries.

The nutritional property of food pertains to a well-balanced ratio of the essential nutrients, carbohydrates, fat, proteins, minerals, and vitamins in accordance with the nutrient requirements of the consumer. Seeds are nutritious, healthy foods that can raise nutritional effectiveness in malnourished and developing parts of the world [28]. Therefore, as the variety of processing methods increases, the question arises as to what influence these methods have on the final product, and there is a concurrent need to assess seed quality for the development of further products. We evaluated seeds before and after oil extraction via four different methods: extraction with subcritical CO_2_, extraction with supercritical CO_2_, solvent (hexane) extraction, and cold pressing. The differences in the nutrient contents are shown in Table 3.

The data presented in Table 3 provide a detailed comparative analysis of the nutrient contents in raspberry seeds before and after different oil extraction methods, including hexane, cold pressing, subcritical CO_2_, and supercritical CO_2_. Each parameter offers insight into how extraction techniques impact the nutritional profile of the seeds. 

Humidity, or moisture content, varies across the methods. The initial moisture content was 5.9%, which increased to 8.6% after hexane extraction and decreased after cold pressing to 4.7%. Both CO_2_ extraction methods resulted in slight increases, suggesting that these methods may maintain or slightly increase moisture, which is possibly due to less heat generation compared to other methods. There is a clear trend of increasing protein content with CO_2_ extraction methods, particularly subcritical (10.1%), which shows the highest protein levels, followed by supercritical (9.4%). The increase in nitrogen percentage supports this finding, as nitrogen is a major component of amino acids in proteins. Cold pressing also increases protein content compared to hexane extraction and the original seed state. Fiber content is highest in seeds processed by subcritical and supercritical CO_2_ extraction (23.9% and 23.1%, respectively), indicating that these methods are beneficial for retaining or even increasing fiber content. This could be due to the gentle nature of CO_2_ extraction, which might preserve the structural components of the seeds better than other methods.

As expected, all extraction methods reduce the fat content of the seeds, with subcritical and supercritical CO_2_ extraction showing the most significant reduction. This suggests effective fat removal in these methods. Cold pressing leaves more residual fat (5.0%) compared to hexane extraction (2.0%), which might be due to less efficiency in fat removal or a less intensive extraction process. All methods result in a decrease in sugar content with the lowest sugar levels observed in seeds processed by supercritical CO_2_ extraction (0.6%). This might be indicative of sugar breakdown or removal during the extraction process.

Regarding seed nutrient content, the processing method was found to have a significant effect on all the research parameters analyzed. Obviously, cold-pressed raspberry seeds have the most remaining fat (5.0%) and sugar (1.4%), so these should be more attractive to the consumer. As an essential component of many compounds found in living plant cells and a key factor determining the productivity of crops for food, feed, fiber, and bio-energy (and hence for all human activities [29]), the nitrogen content varied from 1.2% in the seeds before oil extraction to 1.6% in seeds after subcritical CO_2_ extraction. 

Another parameter that we analyzed was fiber, the daily intake of which should be about 2.4 g per person [30]. Current dietary guidelines recommend an increased dietary fiber intake and suggest that fiber, independent of fat intake, is an important dietary component in preventing some diseases. Recommendations for adult dietary fiber intake generally fall in the range of 20 to 35 g per day [31]. Numerous studies on dietary fiber have proven that this component can prevent and treat some diseases. Diets rich in fiber reduce the risk of certain cancers (large intestine), coronary heart disease (CHD), atherosclerosis, diabetes, and obesity. Additionally, dietary fiber increases fecal bulk, stimulates intestinal peristalsis, and lowers the levels of both total cholesterol and low-density lipoprotein cholesterol in serum. Adding dietary fibers to food is becoming more and more popular [32]. Our research revealed that raspberry seeds are an excellent source of fiber. Before oil pressing, the crude fiber content was 22.1%; after pressing, it ranged from 19.3 to 23.9%. 

Górecka et al. [33] and Saric et al. [34] valorized raspberry seeds as ingredients for gluten-free cookies. The enrichment of gluten-free cookies with raspberry and blueberry fiber concentrates resulted in products with dietary fiber contents higher than 6 g per 100 g, thus making them candidates for bearing “high-fiber” nutritional claims. Our research showed that raspberry seeds, after both supercritical and super-critical CO^2^ extractions, are perfectly suitable for baking. However, cold-pressed seeds need to be ground again, and hexane-extracted seeds cannot be used in baking at all. 

In recent years, plant protein has attracted more attention because of its wide range of sources, short production cycle, and low price [35,36,37]. In our study, it was found that the protein content increased slightly from 7.4 to 10.1% after subcritical CO_2_ seed oil extraction. Therefore, it can be said that the seeds obtained after subcritical CO_2_ extraction are of better quality due to their higher protein content.

The results show that the methods of extraction significantly influence the nutritional composition of raspberry seeds. CO_2_ extraction methods, both subcritical and supercritical, seem to preserve or enhance beneficial nutrients like protein and fiber while effectively reducing fat content. The increase in protein and fiber content post-extraction suggests that the residual seed meal could be used as a valuable dietary supplement or functional food ingredient, particularly in high-protein and high-fiber formulations.

The study results also reveal how extraction methods affect nutrient profiles, which can help manufacturers choose the appropriate technology based on the desired product specifications, such as high-protein supplements or high-fiber food products. Methods that enhance or retain valuable nutrients while minimizing waste (like CO_2_ extractions) align with sustainable production goals, potentially offering lower environmental impacts than more traditional methods like hexane extraction.

The content of macroelements, P, K, Ca, and Mg, and as well as microelements, B, Zn, Cu, Fe, and Mn, were determined in the tested ‘*Polka*’ variety. The data presented in Table 4 offers a detailed analysis of the macro- and micro-nutrient content changes in raspberry seeds. Each of processing methods appears to have a distinct impact on the levels of essential minerals in the seeds. 

Phosphorus levels remain stable at 0.2% after hexane extraction and increase to 0.3% following cold pressing and both CO_2_ extraction methods. This suggests that CO_2_ extraction and cold pressing are effective in preserving or even increasing phosphorus content, which is vital for energy production and bone health. Potassium levels show a slight increase in most extraction methods except for supercritical CO_2_, which returns to baseline levels. This mineral is crucial for cardiovascular health and cellular function. The calcium content slightly fluctuates but generally remains consistent across all methods. Calcium is essential for bone health and metabolic functions. Magnesium content is relatively stable across all methods, showing minor fluctuations but remaining mostly consistent. Magnesium plays a critical role in over 300 enzymatic reactions in the human body. Boron levels nearly double with hexane extraction and cold pressing. This trace element is known for improving bone density and cognitive performance. A dramatic increase in copper content is observed after hexane extraction, which could be due to contamination or the solvent’s interaction with copper present in the seeds. Copper is important for iron metabolism and the functioning of the nervous system. Iron content increases across all extraction methods, particularly after cold pressing. Iron is a crucial component of hemoglobin, which transports oxygen in the blood. Manganese shows a consistent increase after all extraction methods with the highest levels observed after supercritical CO_2_ extraction. Manganese is vital for bone formation, blood clotting, and reducing oxidative stress.

The analyzed samples were also characterized by high levels of zinc (Zn) (from 17.4 mg/kg in the seeds before oil extraction to 27.0 mg/kg in those after extraction with hexane). Due to the number of processes, they are involved in, as well as the continuous research highlighting the benefits of their adequate and balanced intake, it is now known that dietary minerals raise concerns for health specialists and consumers. For example, potassium regulates the heartbeat, assists in muscle concentration, and is needed to send nerve impulses and release energy from fat, carbohydrates, and protein. Calcium is essential for bone and tooth formation. Because of this, calcium requirements are higher during adolescence. Calcium is also very important during later adulthood, which has significant consequences from a public health perspective because an inadequate intake of calcium may increase the risk of osteoporosis [38].

The data suggest that both CO_2_ extraction methods are particularly effective at either maintaining or increasing the content of several essential nutrients in raspberry seeds. Cold pressing also tends to increase the levels of certain minerals like iron and manganese, potentially making the residual seed meal highly nutritious. The unusually high increase in copper content following hexane extraction raises concerns about possible contamination or the nature of chemical interactions during the extraction process. This warrants further investigation to ensure the safety and purity of the extract.

## 4. Conclusions

The quality characteristics of raspberry seeds, both before and after oil extraction using different methods, were revealed for sustainable product development. The seed characteristics varied significantly according to the different processing technologies. The differences in the conditions of these techniques have a noticeable effect on the amount of trace elements in the seeds, affecting their overall nutritional composition. After cold oil extraction or supercritical or subcritical CO_2_ extractions, raspberry seeds were characterized by high nutritional contents of micro- and macroelements.

The study effectively demonstrates that extraction method choice is crucial in determining the nutritional quality of raspberry seed by-products with CO_2_ extraction methods showing promise for producing nutrient-rich, low-fat seed meals suitable for various applications in the food and health industry.

The choice of extraction method significantly affects the mineral content of raspberry seeds, with each method offering different advantages in terms of nutrient retention and potential application in health-oriented products. This provides valuable insights for the food and supplement industries in developing products that leverage the health benefits of raspberry seed by-products.

The results obtained in this study show that seeds, as a by-product of the secondary processing of berries, can be used as a potential functional food and as a high-fiber ingredient in baking. Dried and/or molded raspberry seed by-products are considered potential functional food or pharmaceutical ingredients. They can be used as dietary supplements, for replacing flour in confectionary, for decorating baked goods, and in the production of smoothies and other vegetarian and vegan drinks. Enriched with micro and macroelements, such products would be of high nutritional value. Raspberry seeds after oil extraction would be useful in cooking for athletes, as they are rich in micro and macro-nutrients, dietary fiber and, above all, protein. The use of the pomace in the production of protein bars would be beneficial for both researchers and consumers.

This research shows that raspberry seeds represent a potential source of natural food ingredients. This constitutes the first study to compare the chemical composition of raspberry seeds before and after oil extraction and show the influence of the various processing methods on potential production. By-products from raspberry oil processing industries could be used as a source of technologically and nutritionally distinctive dietary fiber in order to fabricate foods with enhanced nutritional value. 

This research underscores the potential of raspberry seeds as a sustainable and functional food ingredient. By highlighting the methods that best preserve or enhance the quality characteristics of these seeds, the study provides crucial guidelines for industries looking to implement sustainable practices while offering health benefits. The findings encourage continued exploration into the diverse applications of raspberry seed by-products, promoting their inclusion in food products and dietary supplements that contribute positively to consumer health and environmental sustainability.

## Figures and Tables

**Table 1:** Raspberry seeds quality characteristics before oil extraction.

Quality Parameters	Values
Weight	1000 seeds = 2.17 g
Peroxide value	5.64 mEq/kg
Moisture	9.2%
Nutritional energy value	475.25 Kcal

**Table 2:** Raspberry seed colors before and after oil extraction.

	L*	a*	b*	C*	*h*
Raspberry seeds	44.65 ± 1.04 b	12.65 ± 0.45 a	13.26 ± 1.43 b	18.35 ± 0.77 a	46.23 ± 4.01 c
Raspberry seeds after CO_2_ subcritical extraction	45.63 ± 1.93 b	11.54 ± 0.67 a	19.06 ± 1.58 a	22.29 ± 1.70 a	58.78 ± 0.61 b
Raspberry seeds after CO_2_ supercritical extraction	45.81 ± 1.96 b	11.51 ± 0.65 a	19.11 ± 1.59 a	22.27 ± 1.69 a	58.81 ± 0.62 b
Raspberry seeds after cold pressing	55.66 ± 4.78 a	7.72 ± 0.51 b	17.71 ± 1.73 a	19.33 ± 1.64 a	66.35 ± 2.31 a

Note: data are expressed as average value standard deviations of three replicates and different lowercase letters indicate significant differences (*p* < 0.05).

**Table 3:** The nutrient contents (per 100 g) of raspberry seeds before and after oil extraction.

Research Parameter	RS before Oil Pressure	RS after Extraction with Hexane	RS after Cold Pressing of Oil	RS after Subcritical Extraction of CO_2_	RS after Supercritical Extraction of CO_2_
Humidity %	5.9 ± 0.30 c	8.6 ± 0.43 a	4.7 ± 0.24 d	6.0 ± 0.30 bc	6.9 ± 0.34 b
Nitrogen (N) %	1.2 ± 0.06 c	1.2 ± 0.06 c	1.4 ± 0.07 b	1.6 ± 0.08 a	1.5 ± 0.08 ab
Proteins %	7.4 ± 0.37 c	7.6 ± 0.38 c	8.8 ± 0.44 b	10.1 ± 0.50 a	9.4 ± 0.47 ab
Crude fiber content %	22.1 ± 1.11 ab	21.6 ± 1.08 ab	19.3 ± 0.97 b	23.9 ± 1.20 a	23.1 ± 1.15 a
Crude fat and oil content %	11.0 ± 0.55 a	2.0 ± 0.1 c	5.0 ± 0.25 b	0.8 ± 0.04 d	0.9 ± 0.04 d
Sugar %	2.8 ± 0.14 b	0.9 ± 0.05 c	1.4 ± 0.09 a	0.9 ± 0.05 c	0.6 ± 0.03 d

Note: data are expressed as average value standard deviations of three replicates and different lowercase letters indicate significant differences (*p* < 0.05).

**Table 4:** Contents of macro- and micro-components in raspberry seeds before and after oil extraction.

Research Parameter	RS before Oil Pressure	RS after Extraction with Hexane	RS after Cold Pressing of Oil	RS after Subcritical Extraction of CO_2_	RS after Supercritical Extraction of CO_2_
Phosphorus %	0.2 ± 0.01 c	0.2 ± 0.01 c	0.3 ± 0.01 b	0.3 ± 0.02 a	0.3 ± 0.02 a
Potassium (K)	0.2 ± 0.01 c	0.3 ± 0.01 ab	0.3 ± 0.01 a	0.3 ± 0.01 ab	0.2 ± 0.01 bc
Calcium content (Ca) %	0.2 ± 0.01 c	0.1 ± 0.01 c	0.2 ± 0.01 a	0.2 ± 0.01 b	0.2 ± 0.01 b
Magnesium content (Mg) %	0.1 ± 0.00 b	0.1 ± 0.01 b	0.1 ± 0.01 a	0.1 ± 0.01 a	0.1 ± 0.00 c
Boron (B) mg/kg	12.4 ± 0.62 b	26.2 ± 1.31 a	27.9 ± 1.4 a	12.1 ± 0.61 b	11.2 ± 0.56 b
Zinc content (Zn) mg/kg	17.4 ± 0.87 c	27.0 ± 1.35 a	20.9 ± 1.05 b	26.6 ± 1.33 a	26.0 ± 1.30 a
Copper content (Cu) mg/kg	5.1 ± 0.25 b	158.8 ± 7.94 a	6.3 ± 0.31 b	6.3 ± 0.31 b	5.8 ± 0.29 b
Iron content (Fe) mg/kg	29.2 ± 1.46 b	33.8 ± 1.69 b	43.4 ± 2.17 a	41.3 ± 2.07 a	40.8 ± 2.04 a
Manganese content (Mn) mg/kg	45.3 ± 2.27 b	48.1 ± 2.41 b	57.4 ± 2.87 a	60.9 ± 3.05 a	61.8 ± 3.09 a

Note: data are expressed as average value standard deviations of three replicates and different lowercase letters indicate significant differences (*p* < 0.05).

## Data Availability

The original contributions presented in the study are included in the article, further inquiries can be directed to the corresponding author.

## References

[1] Frías-Moreno M.N., Parra-Quezada R.A., González-Aguilar G., Ruíz-Canizales J., Molina-Corral F.J., Sepulveda D.R., Salas-Salazar N., Olivas G.I. (2021). Quality, Bioactive Compounds, Antioxidant Capacity, and Enzymes of Raspberries at Different Maturity Stages, Effects of Organic vs. Conventional Fertilization. Foods.

[2] Krivokapić S., Vlaović M., Damjanović Vratnica B., Perović A., Perović S. (2021). Biowaste as a Potential Source of Bioactive Compounds—A Case Study of Raspberry Fruit Pomace. Foods.

[3] Vulić J., Velićanski A., Ćetejević Simin D., Tumbas Šaponjac V., Djilas S., Cvetković D., Markov S. (2014). Antioxidant, antiproliferative and antimicrobial activity of freeze-dried raspberry. Acta Period. Technol..

[4] Zhang X., Ahuja J.K.C., Burton-Freeman B.M. (2019). Characterization of the nutrient profile of processed red raspberries for use in nutrition labeling and promoting healthy food choices. Nutr. Healthy Aging.

[5] Schulz M., Chim J.F. (2019). Nutritional and bioactive value of Rubus berries. Food Biosci..

[6] Piwowarek K., Lipińska E., Kieliszek M. (2023). Reprocessing of side-streams towards obtaining valuable bacterial metabolites. Appl. Microbiol. Biotechnol..

[7] Alba K., Campbell G.M., Kontogiorgos V. (2019). Dietary fibre from berry processing waste and its impact on bread structure: A review. J. Sci. Food Agric..

[8] Grzelak-Błaszczyk K., Karlińska E., Grzęda K., Rój E., Kołodziejczyk K. (2017). Defatted strawberry seeds as a source of phenolics, dietary fiber and minerals. LWT.

[9] De Souza V.R., Pereira P.A.P., Da Silva T.L.T., De Oliveira Lima L.C., Pio R., Queiroz F. (2014). Determination of the bioactive compounds, antioxidant activity and chemical composition of Brazilian blackberry, red raspberry, strawberry, blueberry and sweet cherry fruits. Food Chem..

[10] Wang S., Zhao F., Wu W., Lyu L., Li W. (2023). Proteins from Blackberry Seeds: Extraction, Osborne Isolate, Characteristics, Functional Properties, and Bioactivities. Int. J. Mol. Sci..

[11] Sławińska N., Prochoń K., Olas B. (2023). A Review on Berry Seeds—A Special Emphasis on Their Chemical Content and Health-Promoting Properties. Nutrients.

[12] Brewer M.S. (2011). Natural Antioxidants: Sources, Compounds, Mechanisms of Action, and Potential Applications. Compr. Rev. Food Sci. Food Saf..

[13] Skrovankova S., Sumczynski D., Mlcek J., Jurikova T., Sochor J. (2015). Bioactive Compounds and Antioxidant Activity in Different Types of Berries. Int. J. Mol. Sci..

[14] Cristobal J., Caldeira C., Corrado S., Sala S. (2018). Techno-economic and profitability analysis of food waste biorefineries at European level. Bioresour. Technol..

[15] (2013). Liu RH, Health-promoting components of fruits and vegetables in the diet. Adv. Nutr..

[16] Brodowska A. (2017). Raspberry Pomace—Composition, Properties and Application. Eur. J. Biol. Res..

[17] Singh B., Pavithran N., Rajput R. (2023). Effects of Food Processing on Nutrients. Curr. J. Appl. Sci. Technol..

[18] ISTA Rules 2024. https://www.seedtest.org/en/publications/international-rules-seed-testing.html.

[19] Ward D., Marini R., Byers R. (2001). Relationships among Day of Year of Drop, Seed Number, and Weight of Mature Apple Fruit. HortScience Publ. Am. Soc. Hortic. Sci..

[20] Sera B., Sery M. (2004). Number and weight of seeds and reproductive strategies of herbaceous plants. Folia Geobot..

[21] Dimić E.B., Vujasinovic V., Radočaj O.F., Pastor O.P. (2012). Characteristics of blackberry and raspberry seeds and oils. Acta Period. Technol..

[22] Van Hoed V., De Clercq N., Echim C., Andjelkovic M., Leber E., Dewettinck K., Verhé R. (2009). Berry seeds: A source of specialty oils with high content of bioactives and nutritional value. J. Food Lipids.

[23] Raczyk M., Bryś J., Brzezińska R., Ostrowska-Ligęza E., Wirkowska-Wojdyła M., Górska A. (2021). Quality assessment of coldpressed strawberry, raspberry and blackberry seed oils intended for cosmetic purposes. Acta Sci. Pol. Technol. Aliment..

[24] Hay F.R., Rezaei S., Wolkis D., McGill C. (2023). Determination and control of seed moisture. Seed Sci. Technol..

[25] Ispiryan A., Bobinaite R., Urbonaviciene D., Sermuksnyte-Alesiuniene K., Viskelis P., Miceikiene A., Viskelis J. (2023). Physico-Chemical Properties, Fatty Acids Profile, and Economic Properties of Raspberry (*Rubus idaeus* L.) Seed Oil, Extracted in Various Ways. Plants.

[26] Oomah B.D., Ladet S., Godfrey D.V., Liang J., Girard B. (2000). Characteristics of raspberry (*Rubus idaeus* L.) seed oil. Food Chem..

[27] Saiedirad M.H., Tabatabaeefar A., Borghei A., Mirsalehi M., Badii F., Ghasemi Varnmakhasti M. (2008). Effect of moisture content; seed size; loading rate and seed orientation on force and energy required for fracturing cumin seed (*Cuminum cyminum* Linn.) under quasi-static loading. J. Food Eng..

[28] Abiola Oso A., Omotayo Ashafa A. (2021). Nutritional Composition of Grain and Seed Proteins.

[29] Sutton M.A., Howard C.M., Erisman J.W.l., Sutton M.A., Howard C.M., Erisman J.W. (2011). Assessing our nitrogen inheritance. The European Nitrogen Assessment.

[30] Górska-Warsewicz H., Kaczorowska J., Laskowski W. (2021). Nutritional Significance of Fruit and Fruit Products in the Average Polish Diet. Nutrients.

[31] Vicente A., Manganaris G., Sozzi G., Crisosto C. (2009). Nutritional Quality of Fruits and Vegetables.

[32] Bowen-Forbes C.S., Zhang Y., Nair M.G. (2010). Anthocyanin content, antioxidant, anti-inflammatory and anticancer properties of blackberry and raspberry fruits. J. Food Compos. Anal..

[33] Górecka D., Pachołek B., Dziedzic K., Górecka M. (2010). Raspberry pomace as a potential fiber source for cookies enrichment. ACTA Scien Tiarum Pol. Technol. Aliment..

[34] Šarić B., Dapčević-Hadnađev T., Hadnađev M., Sakač M., Mandić A., Mišan A., Škrobot D. (2019). Fiber concentrates from raspberry and blueberry pomace in gluten-free cookie formulation: Effect on dough rheology and cookie baking properties. J. Texture Stud..

[35] Ahnen R.T., Jonnalagadda S.S., Slavin J.L. (2019). Role of plant protein in nutrition, wellness, and health. Nutr. Rev..

[36] Liu W.N., Zou M.M., Wang Y.S., Cao F.L., Su E.Z. (2021). Ginkgo Seed Proteins: Characteristics, Functional Properties and Bioactivities. Plant Foods Hum. Nutr..

[37] Sa A.G.A., Moreno Y.M.F., Carciofi B.A.M. (2020). Plant proteins as high-quality nutritional source for human diet. Trends Food Sci. Technol..

[38] Vahapoglu B., Erskine E., Gultekin Subasi B., Capanoglu E. (2021). Recent Studies on Berry Bioactives and Their Health-Promoting Roles. Molecules.

